# Single-Cell Transcriptome Profiling Reveals the Suppressive Role of Retinal Neurons in Microglia Activation Under Diabetes Mellitus

**DOI:** 10.3389/fcell.2021.680947

**Published:** 2021-08-09

**Authors:** Yuhua Xiao, Xing Hu, Shuxin Fan, Jiawei Zhong, Xinzhi Mo, Xialin Liu, Youjin Hu

**Affiliations:** Zhongshan Ophthalmic Center, State Key Laboratory of Ophthalmology, Sun Yat-sen University, Guangzhou, China

**Keywords:** diabetic retinopathy, cell-cell interaction, microglia, neurodegenaration, scRNA seq

## Abstract

Diabetic retinopathy, as one of the common complications of diabetes mellitus, is the leading cause of blindness in the working-age population worldwide. The disease is characterized by damage to retinal vasculature, which is associated with the activation of retina microglial and induces chronic neurodegeneration. Previous studies have identified the effects of activated microglial on the retinal neurons, but the cellular and molecular mechanisms underlying microglial activation is largely unknown. Here, we performed scRNA-seq on the retina of non-human primates with diabetes mellitus, and identified cell-type-specific molecular changes of the six major cell types. By identifying the ligand-receptor expression patterns among different cells, we established the interactome of the whole retina. The data showed that TNF-α signal mediated the activation of microglia through an autocrine manner. And we found TGFβ2, which was upregulated in cone dramatically by hyperglycemia, inhibited microglia activation at the early stage of diabetic retinopathy. In summary, our study is the first to profile cell-specific molecular changes and the cell-cell interactome of retina under diabetes mellitus, paving a way to dissect the cellular and molecular mechanisms underlying early-stage diabetic retinopathy.

## Introduction

Diabetic retinopathy (DR) is a microvascular complication of diabetes mellitus and the leading causing of blindness in working-age population (Wong et al., 2016; Antonetti et al., 2021). DR can be classified into non-proliferation diabetic retinopathy (NPDR) or advanced, proliferation diabetics retinopathy (PDR) (Wong et al., 2016). Vascular endothelial growth factor A (VEGFA) is an effective treatment target for DR. However, anti-VEGF therapy is only effective in the late-stage PDR, and some patients are unresponsive to anti-VEGFA therapy (Ajlan et al., 2016; Park et al., 2017). Understanding the molecular mechanisms underlying DR at early-stage NPDR will reveal opportunities for developing preventive interventions for the earlier stage.

Diabetes mellitus-associated hyperglycemia alters cell-cell interactions in the retina and disrupts retinal homeostasis (Antonetti et al., 2021). Such a disruption in cell-cell interaction causes neuronal apoptosis and damage to the blood-retinal barrier (BRB), which consequently increases retinal vascular permeability and vision loss (Eshaq et al., 2017). Recently studies revealed that inflammatory factors associated with the pathogenesis of diabetic retinopathy (Arima et al., 2020). Pro-inflammatory factors are primarily derived from microglia in the central nervous system. Microglia are activated in numerous degeneration diseases, including Alzheimer’s disease (AD) (Hickman et al., 2018), by various environmental stimuli, such as danger signals and pro-inflammatory factors. Danger signals are recognized by Toll-like receptors (TLR), whereas pro-inflammatory factors are recognized by cognate receptors (Kaminska et al., 2016). Activation of these receptors induces cellular signal transduction via several intracellular pathways such as MAPKs, NF-κB, STATs, and PI3K, which altered the activity of transcription factors and the pattern of global gene expression (Popiolek-Barczyk and Mika, 2016). Microglia activation and increased production of pro-inflammatory factors are associated with early diabetic retinopathy. The coordinative action of reactive oxygen species (ROS) and reactive nitrogen species (RNS), and inflammatory factors damages of BRB and retinal cells (Kinuthia et al., 2020). For instance, high expression of complement proteins in the activated microglia could contribute to retinal cells apoptosis. Microglia secreted inflammatory factors also activate other glial cells, thus further amplifying neuroinflammation (Abcouwer, 2017). Also, the disruption of cross-talk between microglia and the retinal neurons mediates the pathogenesis of the diabetic retinopathy (Altmann and Schmidt, 2018). However, the cellular and molecular mechanisms underlying microglial activation in the early stage of diabetic retinopathy is largely unknown.

Here, we report a single-cell transcriptomic atlas for the retina of cynomolgus monkeys (*Macaca fascicularis*) with diabetes mellitus. Cell-type-specific transcriptional changes associated with hyperglycemia have been revealed. Microglia cells exhibited the most susceptibility to hyperglycemia, characterized by the highest numbers of differentially expressed genes (DEGs) and the most significant alteration of cell-cell communications. Communications between microglia and retinal cells relating to inflammation activation and suppression were also identified, enabling dissection of molecular mechanisms underlying microglia activation under diabetic retinopathy.

## Materials and Methods

### Animal Handling

All animal procedures were under an experimental protocol approved by Sun Yat-sen University’s Standing Committee on Animals. As described previously, Cynomolgus monkeys with spontaneous type 2 diabetes were screened in southern china, and diagnosed as diabetes with the parameters of fasting glucose and HbA1c levels as described before. Non-diabetic age-matched controls were selected randomly from the colony (Fan et al., 2021). All animals were maintained at 25°C on a 12 h light:12 h dark schedule.

### Tissue Procurement and Single Cell Isolation

Animals were anesthetized and perfused with physiological saline, the whole retinal layer were dissected out, and digested in the solution with papain (10 units/ml, Sigma cat. P3125) and DNase at 37°C for 5 min, and dissociated into single cell suspensions with 0.04% bovine serum albumin (BSA) in PBS solution. Single cell suspensions were diluted at a concentration of 500–1800 cells/uL in 0.04% BSA/Ames for loading into 10× Chromium Single Cell Chips.

### Droplet-Based scRNA-Seq

Single cell libraries were constructed using commercial Chromium 3′ v2 and Gel Bead Kit (10× Genomics) following to the manufacturer’s protocol. Single cells were partitioned into Gel beads in EMulsion (GEMs) in the GemCode instrument followed by cell lysis and barcoded reverse transcription of RNA, amplification. Sequencing libraries were sequenced on Illumina HiSeq 2000.

### Cell Culturing

Microglia cells (BV2) were cultured in DMEM (Dulbecco’s modified Eagle’s medium; Gibco, United States) supplemented with 10% fetal bovine serum (FBS), and passaged with 0.05% Trypsin/EDTA. Microglia cells were activated by treating with 100 ng/mL lipopolysaccharide (LPS, Sigma) for 24 h and subsequently with LPS + TGFB2 (50 ng/mL) or LPS for 24 h.

### Quantitative PCR (qPCR)

Total RNA was extracted using TRIzol reagent (Invitrogen) following to the manufacturer’s protocol. Exactly 1 μg of total RNA was reverse transcribed into cDNA using Reverse Transcription Master Mix (Tiangen). qPCR reactions were performed in 384-well plates on a CFX-384 thermocycler (Bio-Rad) using iTaq Universal SYBR Green Supermix (Vazyme) as illustrated by the manufacturer’s instructions. Primer sequences for the experiment are listed in Supplementary Table 1.

### Computational Methods

#### Alignment and Quantification of the scRNA-Seq Data

Sequencing data for single-cell transcriptomics sequencing data was processed using CellRanger (Version 3.1.0). In brief, the raw sequencing reads were mapping to the reference genome of cynomolgus (genome version 5.0 and annotation release 101). UMI counts were quantified to generate a gene-barcode matrix. The filtered gene-barcode matrices for single cells were analyzed using Seurat (V3.1.0) following the tutorial at https://satijalab.org/seurat/v3.1/integration.html. We retained cells that expressed >500 genes for downstream analysis.

### Cell Type Identification

We integrated two datasets using canonical correlation analysis (CCA) of Seurat and group cells with similar transcriptomics into the same cluster. Briefly, the top 2000 highly variable genes from two datasets were selected, and then normalized and integrated. Next, principal component analysis (PCA) was performed on integrated data. The significant principal components (*p*-value < 1E-5) were selected to construct k-nearest neighbor graphs (*k* = 30) based on the Euclidean distance. Cluster these cells into cluster using the Louvain–Jaccard. Cluster-specific genes were identified using the *FindAllMarker* function in Seurat and applied to annotate cluster through comparison with the well-known markers of retinal cell types.

### Differential Expression Analysis and Enrichment Analysis

To identify the differentially expressed genes (DEGs) between the control group and diabetes group, we compared gene expression levels of each cell types in the two groups using Seurat *FindMarker* function with options: test = “MAST” and logfc.threshold = 0.25. Genes with *p*_val_adj < 0.05 and the absolute value of avg_logFC ≥ 0.5 were considered as DEGs.

Gene ontology analysis of DEGs was performed in R package clusterProfiler (version 3.14), which using annotation information provided by the R package org.Hs.eg.db (version 3.10.0).

### Cell-Cell Communication Analysis

Cell-cell communication analysis was performed using CellChat (Version 0.5), based on the known ligand-receptor pairs in cell types. Briefly, the normalized genes expression matrix and cell labels generated by Seurat acted as input for CellChat. The overexpressed ligands and receptors in each cell types were calculated and then projected into the protein-protein interaction network. Next, to infer the biologically significant cell–cell communication, we assigned each interaction with a probability value and performed a permutation test. Cell-cell communication altered under DM was analyzed by joint manifold learning and quantitative contrasts of multiple cell-cell communication networks. Finally, communication networks were visualized using circle plot and signaling pathways were illustrated by using bubble plot.

### Statistical Analysis

All statistical data were analyzed using R software (version 3.6.0). Statistical significance was calculated with unpaired two-tailed Student *t* test. *P* < 0.05 was considered statistically significant.

### Code and Data Accession

All sequencing data and expression metric for this study are available in Gene Expression Omnibus (GEO) with the accession number GSE168908.

## Results

### Establishing of the Cell Atlas for Cynomolgus Monkey Retina Based on Single-Cell RNA-Seq

Consistent with our previous study, the fasting blood glucose levels was 32.7 mmol/L in spontaneous type 2 diabetes monkey versus 4.46 mmol/L in control monkey, and the glycated hemoglobin (HbA1c) was 11% in diabetes versus 3.8% in control. To elucidate the cell-type-specific gene expression alterations associated with non-proliferative diabetic retinopathy, we isolated the entire neural retina from diabetic and age-matched healthy cynomolgus monkey (*M. fascicularis*), and performed droplet-based scRNA-seq. In total, 10263 high-quality cell profiles were retained for downstream analysis, including 8708 retinal cells from the control and 1555 retinal cells from diabetic monkey.

**FIGURE 1:**
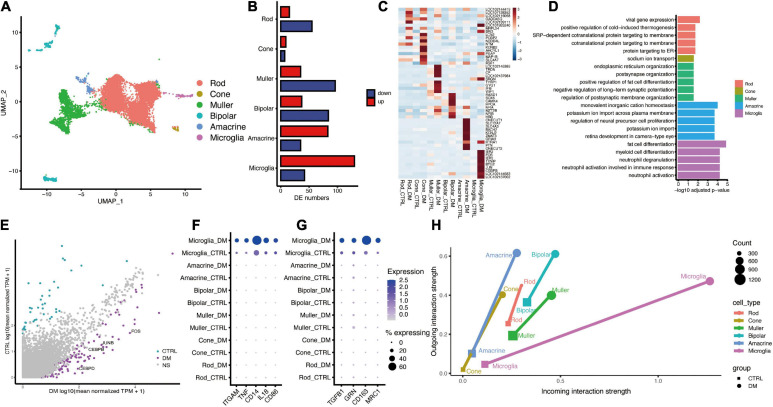
The single-cell atlas and interactome of the monkey retina under diabetes mellitus. **(A)** UMAP plot showing the identified cell types, including Rod, Cone, Muller, Bipolar, Amacrine, and Microglia. **(B)** Bar plot showing the number of DEGs between diabetes and control groups. **(C)** Heatmap showing top (*n* = 10) up-regulated hyperglycemia-associated DEGs in different cell types. **(D)** Representative GO terms of hyperglycemia-induced genes in each cell type. **(E)** Scatter plot showing DEGs of Microglia. Purple and cyan denote genes that are significantly changed under DM. **(F,G)** Dot plot showing the augmented expression of pro-inflammatory genes **(F)** and anti-inflammatory genes **(G)** in microglia under DM. **(H)** Dot plot showing the strength of outgoing and incoming interaction of different cell types under normal and hyperglycemia conditions. The length of the line connecting two dots of the same cell type corresponds to extent of the alteration.

Unbiased clustering identified 13 clusters that existed in both groups (Supplementary Figures 1A,B). We annotated six cell types (Figure 1A), including two glial cell types (microglia and Muller glia cells) and four neuronal cell types (rod and cone photoreceptors, amacrine cells, and bipolar cells) according to the expression pattern of well-known marker genes for each cell type –Rods expressing *SAG*, *NRL*, *GNAT1*, and *RHO*; Cone expressing *GNGT2*, *GUCA1C*, *ARR3*, and *OPN1WM*; Amacrine expressing *TFAP2A*, *TFAP2B*, and *GAD1*; Bipolar expressing *TMEM215*, *VSX2*, and *CABP5*; Muller expressing *APOE*, *GLUL*, *RLBP1*, and *CRYAB*; Microglia expressing *C1QA*, *C1QB*, and *C1QC* (Supplementary Figure 1C). Function annotation of cell-type-specific genes revealed the functional characteristics of non-human primates (NHPs) retinal cells as following: “phototransduction” for Rod and Cone, “Vesicle-mediated transport in synapse” for bipolar and “neutrophil mediated immunity” for microglia (Supplementary Figures 1D,E), which were concurred with the known function of retinal cells.

### Cell-Type-Specific Differential Gene Expression Analysis Revealed Microglial Activation Under Hyperglycemia

Through differentially expression analysis of each retinal cell type, microglia showed the largest number of up-regulated genes under diabetic mellitus (Figures 1B,C), indicating that microglia are significantly affected by hyperglycemia. GO analysis of DEGs revealed that “neutrophil activation,” “regulation of neuron death,” and “epithelial cell migration” were enriched in up-regulated genes within microglia (Figure 1D), indicating that hyperglycemia activated retinal microglia. Some crucial genes related to inflammatory regulation were highly expressed, including *CEBPB*, *CEBPD*, *JUN*, and *FOS* (Figure 1E). *CEBPB* and *CEBPD* belong to CCAAT/enhancer binding protein (c/EBP) family, which is implicated in inflammatory and required for activation of microglia (Straccia et al., 2011), *JUN* and *FOS* formed AP-1 transcription factor, which induces pro-inflammatory cytokine expression (Qiao et al., 2016). Furthermore, we analyzed the marker genes related to pro-inflammatory (M1) and anti-inflammatory (M2) (Leng and Edison, 2020). Both M1 and M2 marker genes were elevated by hyperglycemia (Figures 1F,G), a similar finding was reported in other neuroinflammatory diseases, such as spinal cord injury (Kigerl et al., 2009). Collectively, these results demonstrate that hyperglycemia induces microglial activation and elevates inflammation levels.

### Microglia Activation Is Associated With Enhanced Autocrine TNF-α Signaling Under Hyperglycemia

To reveal the communication between activated microglia and other retinal cells, we performed cell-cell communication analysis using R package CellChat (Jin et al., 2021). The overall cell-cell communication strength was enhanced by hyperglycemia, whereas the largest strength of outgoing and incoming signaling was found in microglia (Figure 1H).

**FIGURE 2:**
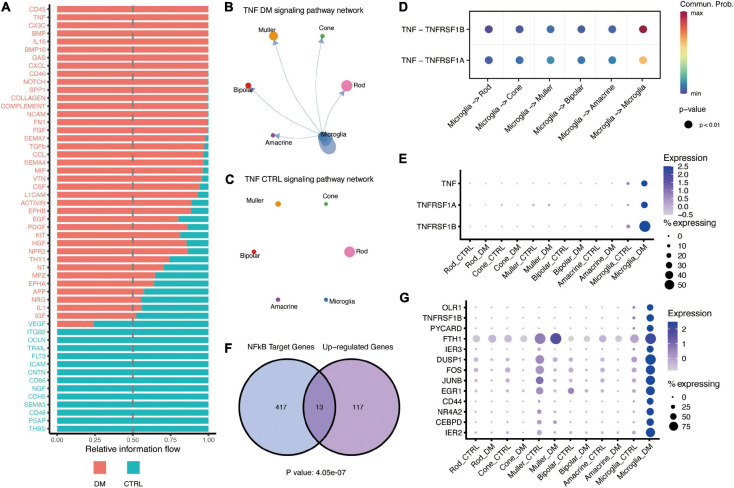
TNF-α mediates interactions between activated microglia and other retinal cells under DM. **(A)** Barplot showing the all signaling pathways received by microglia, which were ranked based on their differences of overall information flow within the inferred networks. The top signaling (shown in red) are more enriched in diabetes, whereas the bottle ones (shown in green) are more enriched in control. **(B,C)** The inferred TNF signaling networks in the retina of DM **(B)** and control **(C)** monkey. Circle sizes are proportional to the number of cells in each cell type, width of the arrow lines represents the communication probability. **(D)** The significant ligand-receptor pairs of the TNF-α signaling pathway. The dot color and size represent the calculated communication probability and *p*-values, respectively. *p*-values are computed from the one-sided permutation test. **(E)** Dot plot showing the expression of ligand TNF-α and its receptors TNFR1 (*TNFRSF1A*) and TNFR2 (*TNFRSF1B*). **(F)** Venn diagram showing the significant enrichment of NF-κB target genes in microglia activated by hyperglycemia. **(G)** Dotplot showing the expression of NF-κB target genes enhanced by hyperglycemia in microglia.

Next, we focused on the signaling pathway sent and/or received by microglia (Figure 2A), including CD45, TNF, THBS, and PSAP. TNF signaling, known pro-inflammatory signaling in multiple diseases, was predicated to mediate the self-communication of microglia (Figures 2B,C). The communication was mediated by ligand TNF-α and its two receptors, TNFR1 and TNFR2, which were elevated by hyperglycemia in microglia (Figures 2D,E). Previous studies had revealed that TNF-α and TNF receptors could trigger the activation of NF-κB to activate the downstream inflammatory genes (Hayden and Ghosh, 2014; Bras et al., 2020). Our analysis demonstrated that the NF-κB targeted genes were significantly enriched in the upregulated genes in the microglia (*P* values < 4.05e-10), including *CEBPD*, *JUNB*, and *FOS*, and *TNFRSF1B* receptor (Figures 2F,G and Supplementary Figures 3A,B). These findings suggested that TNF potentially mediates the activation of NF-κB signaling in an autocrine manner; this consequently activates microglia under hyperglycemia.

### Communication Between Microglia and Retinal Cells Is a Potential Feedback to Suppress the Excessive Activation of Microglia

**FIGURE 3:**
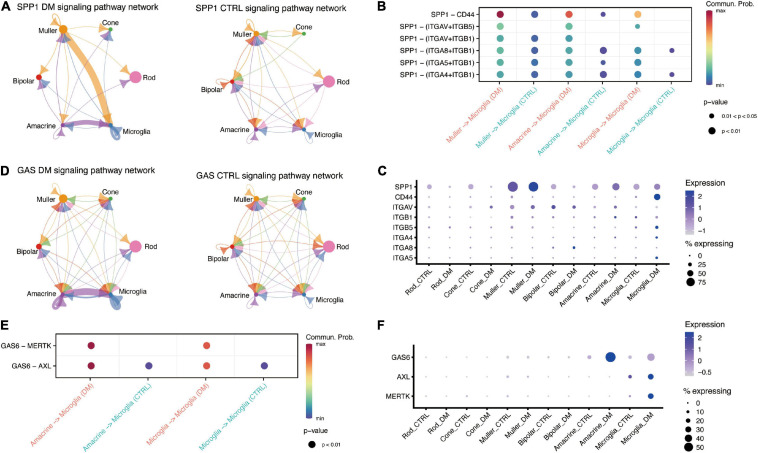
Retinal cells communicate with microglia via SPP1 and GAS6 signaling pathway. **(A)** The inferred SPP1 signaling networks in the retina of DM (left) and control (right) monkeys. **(B)** Comparison of significant ligand-receptor pairs of SPP1 signaling pathway between DM and control retina. **(C)** Dotplot showing the expression of ligand *SPP1* and its receptors, including *CD44*, in DM and control retina. **(D)** The inferred GAS signaling networks between DM (left) and control (right) retina. **(E)** Comparison of significant ligand-receptor pairs of GAS signaling pathway between DM and control. **(F)** Dotplot showing the expression of ligand *GAS6* and its receptors *AXL* and *MERTK*, in DM and control retina.

OPN (SPP1) signaling is also enhanced under hyperglycemia. OPN is a proinflammatory cytokine that regulates the activation and function of microglia (Yu et al., 2017). We found OPN was mainly expressed in both Muller and microglia (Figure 3A), whereas its receptor, CD44, was up-regulated in microglia by hyperglycemia (Figures 3B,C). A previous study have demonstrated that OPN could down-regulation the expression of TNF in microglia (Rabenstein et al., 2016). Our data suggested that OPN secreted by Muller and microglia could suppress TNF activation to impede excessive activation of microglia. Similarly, our data showed that GAS6 mediated cell-cell communication between amacrine and microglia was enhanced by hyperglycemia, and negatively regulated the pro-inflammatory via TAM receptors, Axl and Mer (Gilchrist et al., 2020; Figure 3E). CellChat predicted that hyperglycemia-induced GAS6 signaling was mediated by ligand GAS6 and both two receptors (Figure 3D), Ligand GAS6 was up-regulated in microglia and amacrine, whereas the receptors were up-regulated in microglia (Figure 3F).

**FIGURE 4:**
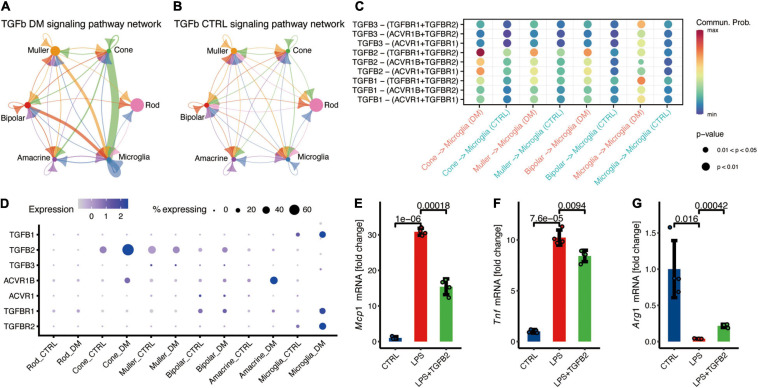
Cone communicate with microglia via TGFβ signaling pathway, potentially suppress the microglia inflammation. **(A,B)** The inferred TGFβ signaling networks in the retina of DM **(A)** and control monkeys **(B)**. **(C)** Comparison of significant ligand-receptor pairs of TGFβ signaling pathway between DM and control retina. **(D)** Expression distribution of TGFβ signaling genes. **(E–G)** Fold change to control based on the relative expression of *Mcp1* **(E)**, *Tnf* **(F)**, and *Arg1* **(G)**. Data are presented as Mean + SD from four individual experiments, *p* values was calculated using two-sided *t* test.

Moreover, we reported enhanced communication between cone and microglia through TGF-β signaling pathways (Figures 4A,B). TGF-β is a major anti-inflammatory factor, which triggers the downstream expression of target genes through TGFBR1 and TGFBR2 receptors. Previous studies confirmed that overexpression of TGFB1 in the cones can reduce the cone degeneration caused by retinitis pigmentosa through microglia (Wang et al., 2020) and ablation of TGFBR2 in retinal microglia lead to the microglia activation and promote the pathological microglial gene expression profile (Ma et al., 2019). Our study revealed that *TGFB2* potentially mediates the communication between cone and microglia under hyperglycemia (Figures 4C,D).

### TGFB2 Attenuated the Expression of Pro-inflammatory Factors in Microglia

To investigate the function of TGFβ signaling in microglia, microglial BV2 cell lines were cultured for *in vitro* recombination. Microglia treated with LPS significantly increased expression of *Tnf* and *Mcp1* (Figures 4E,F), and decreased the expression of *Arg1* (Figure 4G), indicating the inflammation activation in microglia. TGFβ2 treatment resulted in the significantly decreased the LPS-induced *Mcp1* and *Tnf* expression (Figures 4E,F), and augmented *Arg1* expression in microglia (Figure 4G). In summary, these data demonstrated that TGFβ2 suppress microglia activation and potentially reduce the neurotoxicity.

These findings demonstrated that communication between microglia and other retinal cells, including photoreceptors, is a potential feedback mechanism to suppress the excess activation of microglia under diabetes mellitus.

## Discussion

In this study, we have generated a single-cell transcriptomic atlas of the NHP retina under hyperglycemia. Through identification of the ligand-receptor expression patterns among single cells, we assessed the interactome of the whole retina in the diabetes mellitus group and the control group. These findings revealed that microglia activation under hyperglycemia was mediated by autocrine pro-inflammatory factors, including TNF-α. Retinal cells potentially suppressed excessive microglial activation by secreting anti-inflammatory factors, including TGFβ2 secreted by the cone. The present study collectively identified cell-specific molecular changes and the cell-cell interactome of the retina under diabetes mellitus. The findings will pave the way to uncover the cellular and molecular mechanisms underlying early-stage diabetic retinopathy.

Moreover, the results indicated that interaction between retinal cells was significantly altered by hyperglycemia, which potentially contributes to the pathological development of diabetic retinopathy. Hyperglycemia induced significantly high expression of pro-inflammatory factors in microglia, including TNF-α, IL18, etc. So, the activated microglia would affect the retinal neurons through paracrine. In addition, as our data showed that microglia is the major source of TNF-α, which can directly affect microglia through autocrine. Of note, the NF-κB regulated TNF receptor, TNFR2, was upregulated under diabetes mellitus, suggesting the association of NF-κB signaling with the autocrine effects of TNF-α on microglia.

Of note, effects of activated microglia on endothelial during the development of diabetic retinopathy. Evidences from animal model showed that blocking the TNF-α significantly reduced the vascular change induced by diabetes, such as loss of pericyte (Behl et al., 2008; Semeraro et al., 2015). Our previous work observed the microangiopathy with remarkable acellular capillaries and pericyte loss in monkeys with spontaneous DM (Fan et al., 2021). TNF-α increase retinal endothelial permeability by increased the expression of ANG2 and downregulated the expression of tight junction proteins (Kim et al., 2000; Fiedler and Augustin, 2006; Aveleira et al., 2010). The upregulated TNF-α in microglia suggesting that microglia may contribute to the break of blood-retinal barriers through communicating with vascular endothelial cell (van der Wijk et al., 2017). These data showed microglia activation was accompanied with and may even mediate the retinal vascular abnormality, providing a potential target to prevent the progress of diabetic retinopathy. However, this study was limited by lack of a significant number of endothelial, communications between endothelial and retinal cells need to be further investigated.

Our data showed that non-human primates have an autologous mechanism to inhibit microglia activation. Reports show that TGFβ is involved in microglia activation, and mediate neuron protection in many neurodegeneration diseases, such as AD and Parkinson’s disease (PD) (Caraci et al., 2012; Chen et al., 2017). Another study found that TGFβ1 could rescue degenerating cones through a microglia-mediated mechanism (Wang et al., 2020). Cone communicates with microglia through secreted TGFβ2 instead of TGFβ1, suggesting a novel target sites of TGFβ mediating the effects of microglia under diabetes mellitus. In addition, we also found other retinal cells suppress the activation of microglia. For example, amacrine inhibited microglia activation through SPP1 and GAS signaling pathways, and Muller inhibited microglia activation through the GAS signaling pathway. In the non-pathological state, microglia are activated by infection or injury and can exhibit inflammation via self-limited manner to regulate the homeostasis. However, hyperglycemia-induced chronic stress may lead to neuroinflammatory fails to revolve through self-limited manner and leading to the disease.

Consistent with the previous studies, our data showed that the gene expression in the retina is heterogeneous, with obvious cell type specific expression pattern which corresponding to differential functions of different retinal cells (Macosko et al., 2015; Peng et al., 2019). For example, our data showed that TGFB2 is heterogeneously expressed among different retinal cells, and seems specific to the photoreceptors. Multiple factors to regulate gene transcription, including the transcription factors (such as Forkhead Transcription Factors) (Massague et al., 2000) and epigenetic regulations (TGFB2 enhancer status), may result in the heterogeneous expression of the genes among various retinal cells (Samatar et al., 2002; Shin et al., 2019). For example, BRD4 was shown to cooperate with NF-κB to induce TGFB2 expression by activating TGFB2 enhancer (Shin et al., 2019). In this study, hyperglycemia-induced cone-specific expression of NF-κB family members, including REL, RELA, and NFKB2. We anticipate the upregulation of TGFB2 mRNA levels may be due to the increased expression of BRD4 and NF-κB in cone (Supplementary Figure 3C). Future studies integrating single-cell multi-omics data (Hu et al., 2018) including the transcriptome and epigenome may clarify the detailed mechanisms underlying the differential expression among different retinal cells.

In conclusion, the present study affirms that retinal cells potentially limit the activation of microglia through cell-cell interaction, providing a novel insight into the molecular mechanisms of homeostasis maintenance in patients with diabetic retinopathy. Of note, the findings in this study were based on RNA expression data of single cells, the detailed mechanisms should be further studied in the future.

## Data Availability Statement

The datasets presented in this study can be found in online repositories. The names of the repository/repositories and accession number(s) can be found below: https://www.ncbi.nlm.nih.gov/geo/query/acc.cgi?acc=GSE168908.

## Ethics Statement

The animal study was reviewed and approved by the Institutional Animal Care and Use Committee (IACUC) of the Zhongshan Ophthalmic Center of Sun Yat-sen University (2018-171).

## Author Contributions

YH conceived and designed the study. YX, SF, and XH performed the experiments. YH, YX, JZ, and XM analyzed the data and performed statistical analyses. YH, YX, JZ, XL, and XH interpreted the data and wrote the manuscript in discussion with all authors. All authors contributed to the article and approved the submitted version.

## Conflict of Interest

The authors declare that the research was conducted in the absence of any commercial or financial relationships that could be construed as a potential conflict of interest.

## Publisher’s Note

All claims expressed in this article are solely those of the authors and do not necessarily represent those of their affiliated organizations, or those of the publisher, the editors and the reviewers. Any product that may be evaluated in this article, or claim that may be made by its manufacturer, is not guaranteed or endorsed by the publisher.

## Supplementary Material

Supplementary Figure 1 Identification cell types with single-cell transcriptomic profiling. **(A,B)** UMAP plot showing the single-cell distribution in groups **(A)** and clusters **(B)**. **(C)** Dot plot showing the expression of known marker genes across different cell types. **(D)** Heatmaps showing the top-ranked 50 (ranked by the average difference among different cell types) marker genes of each cell type. **(E)** Representative GO terms for each cell type marker genes.

Supplementary Figure 2 Comparison of outgoing and incoming signals in retina of DM and control monkey. All outgoing signaling **(A)** or incoming signaling **(B)** for each cell type in two groups. DM: Diabetes mellitus; CTRL: Control.

Supplementary Figure 3 Activation of NF-κB targets genes which were upregulated in the retina of DM monkey. **(A)** Venn diagram showing the number of NF-κB target genes and up-regulated genes by hyperglycemia in the Rod. **(B)** Dot plot showing the expression of the NF-κB target genes, which are up-regulated by hyperglycemia in Rod. **(C)** Dot plot showing the expression of the NF-κB signals in the retinal cells of DM monkeys.
