# Supplementary figures and images for: Single-Cell Transcriptome Profiling Reveals the Suppressive Role of Retinal Neurons in Microglia Activation Under Diabetes Mellitus
Source: Front Cell Dev Biol. 2021 Aug 9;9:680947. doi: 10.3389/fcell.2021.680947 (PMC8381733; doi:10.3389/fcell.2021.680947)

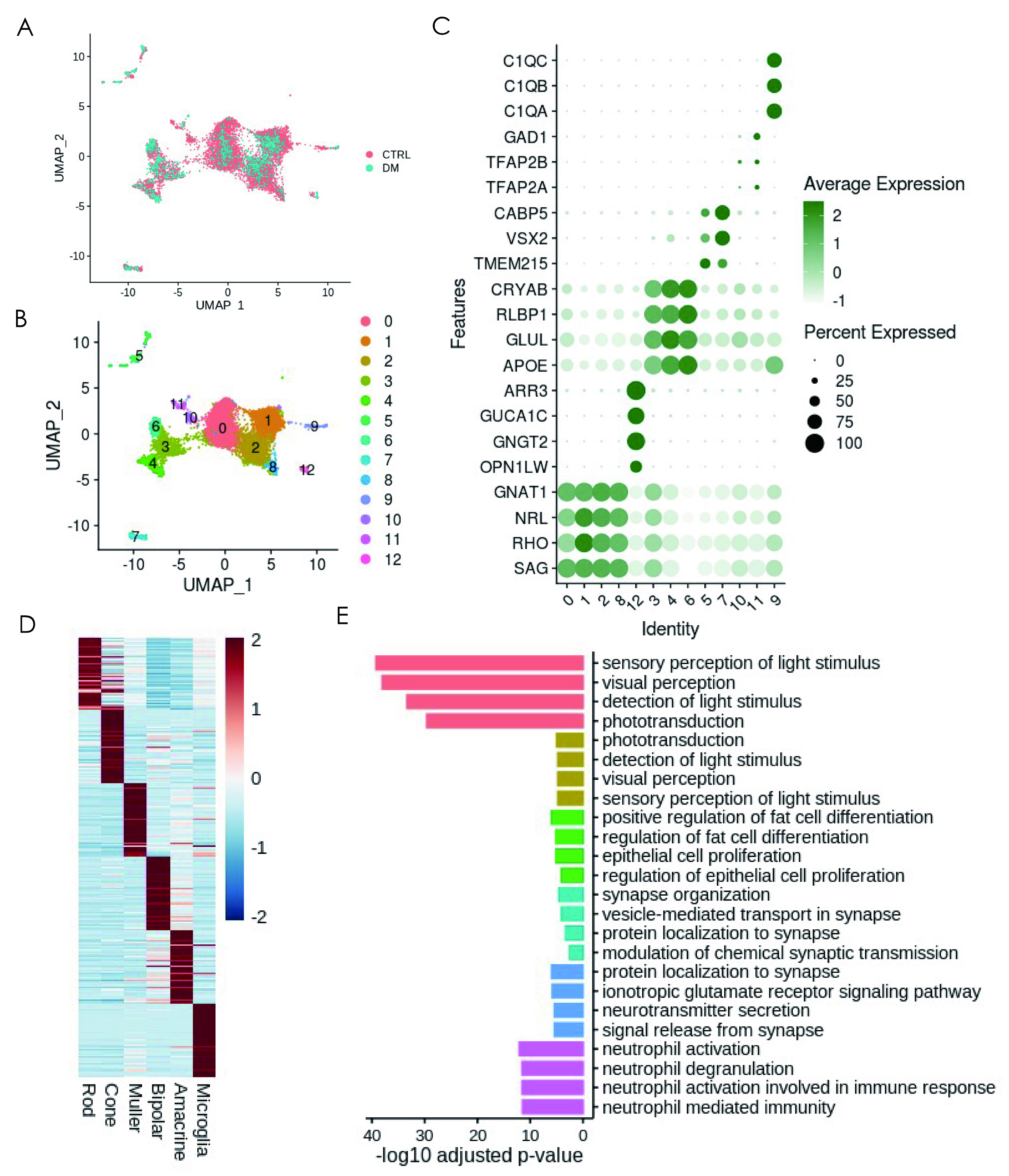

Supplement: Supplementary Figure 1 — Identification cell types with single-cell transcriptomic profiling. (A,B) UMAP plot showing the single-cell distribution in groups (A) and clusters (B). (C) Dot plot showing the expression of known marker genes across different cell types. (D) Heatmaps showing the top-ranked 50 (ranked by the average difference among different cell types) marker genes of each cell type. (E) Representative GO terms for each cell type marker genes. [file Image_1.tif]

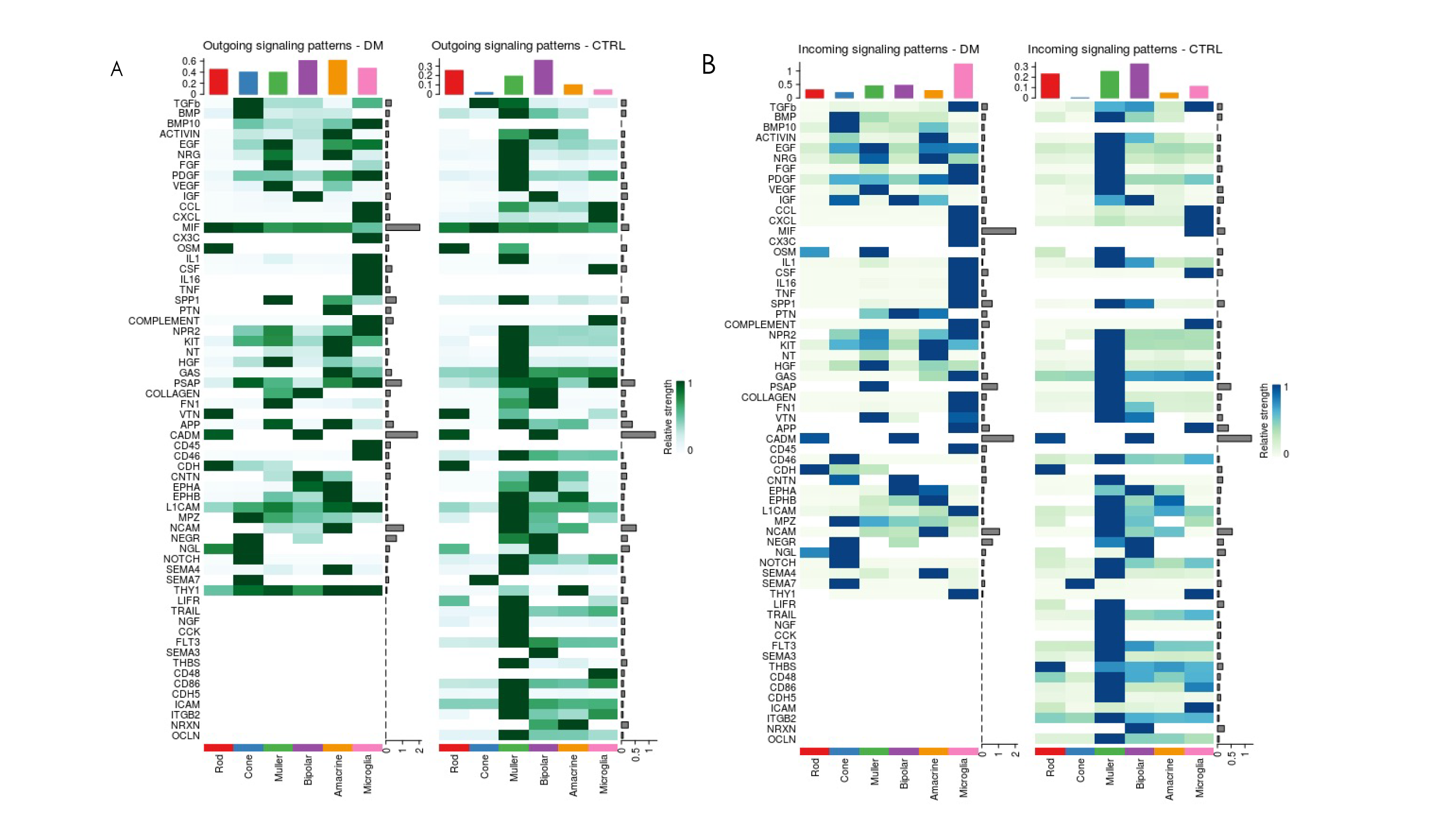

Supplement: Supplementary Figure 2 — Comparison of outgoing and incoming signals in retina of DM and control monkey. All outgoing signaling (A) or incoming signaling (B) for each cell type in two groups. DM: Diabetes mellitus; CTRL: Control. [file Image_2.TIF]

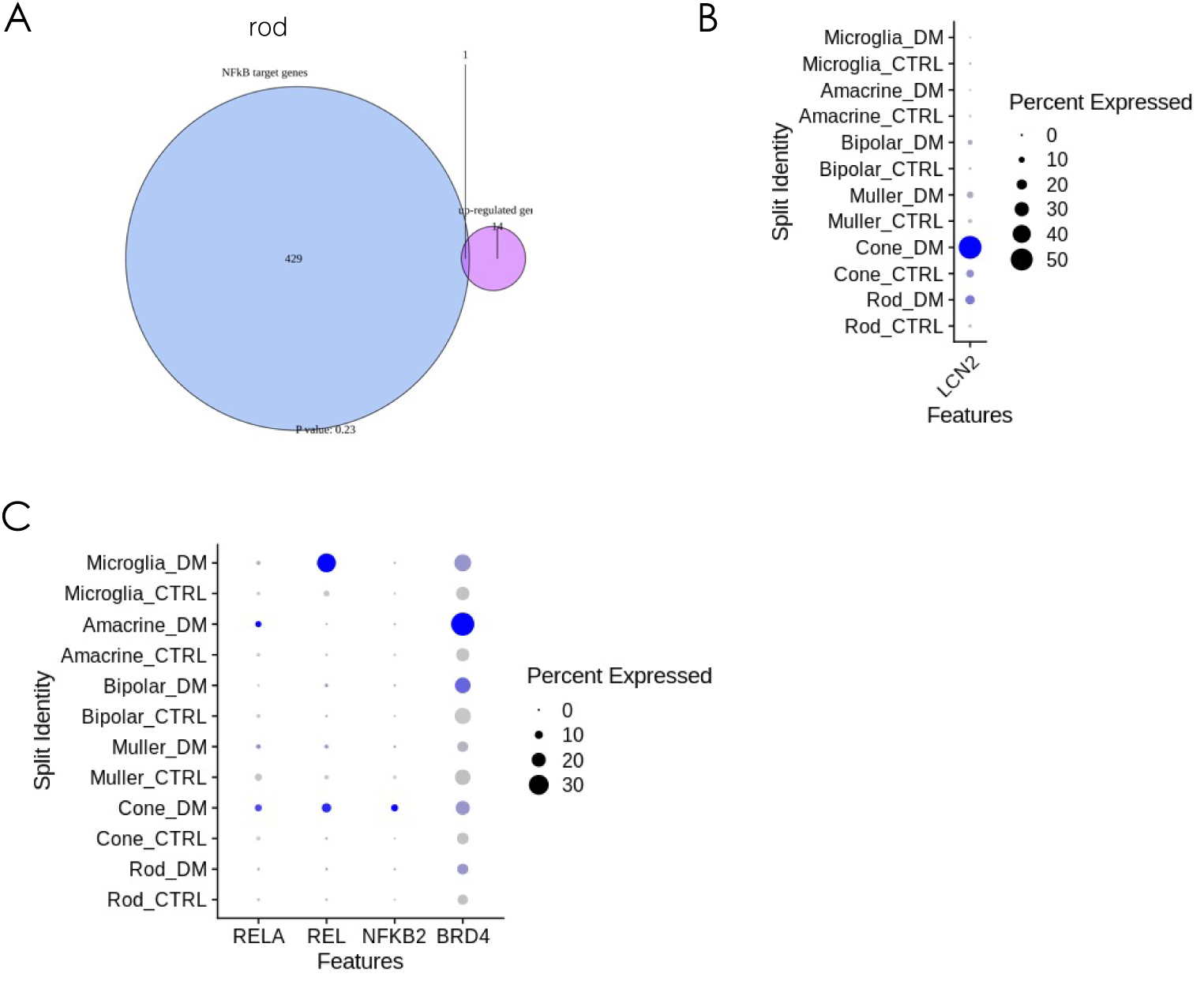

Supplement: Supplementary Figure 3 — Activation of NF-κB targets genes which were upregulated in the retina of DM monkey. (A) Venn diagram showing the number of NF-κB target genes and up-regulated genes by hyperglycemia in the Rod. (B) Dot plot showing the expression of the NF-κB target genes, which are up-regulated by hyperglycemia in Rod. (C) Dot plot showing the expression of the NF-κB signals in the retinal cells of DM monkeys. [file Image_3.TIF]
